# Using surveillance data to estimate pandemic vaccine effectiveness against laboratory confirmed influenza A(H1N1)2009 infection: two case-control studies, Spain, season 2009-2010

**DOI:** 10.1186/1471-2458-11-899

**Published:** 2011-11-30

**Authors:** Camelia Savulescu, Silvia Jiménez-Jorge, Salvador de Mateo, Francisco Pozo, Inmaculada Casas, Pilar Pérez Breña, Antonia Galmés, Juana M Vanrell, Carolina Rodriguez, Tomas Vega, Ana Martinez, Nuria Torner, Julián M Ramos, Maria C Serrano, Jesús Castilla, Manuel García Cenoz, Jone M Altzibar, Jose M Arteagoitia, Carmen Quiñones, Milagros Perucha, Amparo Larrauri

**Affiliations:** 1 Institute of Health Carlos III, National Centre of Epidemiology, c/Monforte de Lemos no.5, 28029 Madrid, Spain; 2 EpiConcept, 47, rue de Charenton 75012 Paris, France; 3 Institute of Health Carlos III, National Centre for Microbiology, National Influenza Centre, 28220 Majadahonda, Madrid, Spain; 4 Service of Epidemiology, General Directorate of Public Health, Baleares, c/Cecilio Metelo 18. 07003 Palma de Mallorca, Spain; 5 Dirección General de Salud Pública e Investigación, Desarrollo e Innovación. Consejería de Sanidad de Castilla y León, Paseo Zorrilla 1, 47071 Valladolid, Spain; 6 Department of Health, Generalitat of Catalonia, C/Roc Boronat 81-95, 08005 Barcelona, Spain; 7 Sub-directorate of Epidemiology, Public Health Directorate, Avda América 2, 06800 Mérida, Badajoz, Spain; 8 Institute of Public Health of Navarra, Leyre 15, 31003 Pamplona, Spain; 9 Sub-directorate of Public Health, Gipuzkoa, Avda Navarra 4, 20013 Donostia-San Sebastián, Spain; 10 Public Health Service, Department of Health, Basque Government, C/Donostia-San Sebastian 1, 01010, Vitoria-Gasteiz, Spain; 11 General Directorate of Public Health and Consumption, Consejería de Salud. La Rioja Government, C/Gran Vía del rey D. Juan Carlos, no 18, 26071 Logroño, Spain; 12 Consortium for Biomedical Research in Epidemiology & Public Health, Institute of Health Carlos III, Spain

## Abstract

**Background:**

Physicians of the Spanish Influenza Sentinel Surveillance System report and systematically swab patients attended to their practices for influenza-like illness (ILI). Within the surveillance system, some Spanish regions also participated in an observational study aiming at estimating influenza vaccine effectiveness (cycEVA study). During the season 2009-2010, we estimated pandemic influenza vaccine effectiveness using both the influenza surveillance data and the cycEVA study.

**Methods:**

We conducted two case-control studies using the test-negative design, between weeks 48/2009 and 8/2010 of the pandemic season. The surveillance-based study included all swabbed patients in the sentinel surveillance system. The cycEVA study included swabbed patients from seven Spanish regions. Cases were laboratory-confirmed pandemic influenza A(H1N1)2009. Controls were ILI patients testing negative for any type of influenza. Variables collected in both studies included demographic data, vaccination status, laboratory results, chronic conditions, and pregnancy. Additionally, cycEVA questionnaire collected data on previous influenza vaccination, smoking, functional status, hospitalisations, visits to the general practitioners, and obesity. We used logistic regression to calculate adjusted odds ratios (OR), computing pandemic influenza vaccine effectiveness as (1-OR)*100.

**Results:**

We included 331 cases and 995 controls in the surveillance-based study and 85 cases and 351 controls in the cycEVA study. We detected nine (2.7%) and two (2.4%) vaccine failures in the surveillance-based and cycEVA studies, respectively. Adjusting for variables collected in surveillance database and swabbing month, pandemic influenza vaccine effectiveness was 62% (95% confidence interval (CI): -5; 87). The cycEVA vaccine effectiveness was 64% (95%CI: -225; 96) when adjusting for common variables with the surveillance system and 75% (95%CI: -293; 98) adjusting for all variables collected.

**Conclusion:**

Point estimates of the pandemic influenza vaccine effectiveness suggested a protective effect of the pandemic vaccine against laboratory-confirmed influenza A(H1N1)2009 in the season 2009-2010. Both studies were limited by the low vaccine coverage and the late start of the vaccination campaign. Routine influenza surveillance provides reliable estimates and could be used for influenza vaccine effectiveness studies in future seasons taken into account the surveillance system limitations.

## Background

In April 2009, Spain reported the first case of pandemic influenza A(H1N1)2009 infection in Europe [1] and since then the pandemic virus activity was monitored by the Spanish Influenza Sentinel Surveillance System (SISSS). The system has been in place since 1996 [2] to provide timely epidemiological and virological information on influenza activity in Spain [3] also participating in the European Influenza Surveillance Network [4]. To better monitor the pandemic influenza, the 17 Spanish regional sentinel networks integrated in the surveillance system increased the number of sentinel general practitioners and paediatricians participating in influenza surveillance and introduced systematic swabbing of patients.

Annually, in Spain, seasonal influenza vaccination is recommended to high risk groups for influenza complications: patients with chronic conditions over six month old, health care workers, and the elderly [5]. The seasonal influenza vaccine 2008-2009 showed no effect in preventing the pandemic influenza A(H1N1)2009 infection [6]. However, the routine vaccination campaign was conducted between September and November 2009 using a seasonal vaccine [7] similar to the 2008-2009 one.

The pandemic vaccination campaign started on 16 November 2009 (week 46/2009) and continued over the season using the World Health Organization recommended pandemic monovalent vaccine based on A/California/7/09-like virus [8]. The vaccine was recommended for health professionals, essential services, any person over six month old with chronic conditions (heart diseases, pulmonary diseases (including asthma), renal, liver, metabolic, neuromuscular, and immune diseases), morbidly obese, pregnant women, and close contacts of high risk groups [9]. Various vaccine brands were used, mainly adjuvanted in most of the risk groups and non-adjuvanted in pregnant women and children.

Since the season 2008-2009, Spain has been participating in the European Centre for Disease Prevention and Control (ECDC) funded project "Influenza-Monitoring of Vaccine Effectiveness" (I-MOVE) [10], aimed at identifying the best method to estimate influenza vaccine effectiveness in the European Union (EU). As part of the I-MOVE project, different designs were piloted among elderly population, to identify the best approach to estimate influenza vaccine effectiveness in Spain (cycEVA study). The test negative design comparing the vaccination status of the laboratory confirmed cases to that of patients testing negative for influenza was considered feasible and adequate for Spain, in the context of a pandemic [11].

The routine surveillance system has been previously used [12-14] to estimate the effectiveness of seasonal vaccine against the circulating influenza strain, using the test-negative design. It was considered that when suffering an episode of influenza like illness (ILI), the test negative controls consult sentinel physicians in the same way as the influenza laboratory confirmed cases, reducing the bias related to health seeking behaviour [15,16].

We aimed to estimate the pandemic influenza vaccine effectiveness (PIVE) against laboratory confirmed pandemic influenza A(H1N1)2009 infection using both the surveillance dataset and the cycEVA study, in order to explore the capacity of surveillance system to provide annually estimates of influenza vaccine effectiveness in Spain.

## Methods

Using the case-control test-negative design during the influenza season 2009-2010, we analysed the influenza surveillance data (surveillance-based study) and we conducted an observational study (cycEVA) embedded in the surveillance system, collecting more information in a better controlled way. We describe the two studies below.

### Surveillance-based study

A total of 647 general practitioners and 220 paediatricians from 17 out of 19 Spanish regions participated in the influenza sentinel surveillance system during the 2009-2010 season, covering 2.6% of the Spanish population. Sentinel physicians systematically swabbed the first two patients presenting with ILI each week. A case definition based on the EU ILI definition [17] was recommended for patient swabbing as follows: sudden onset of symptoms, and at least one systemic symptom (fever or feverishness, malaise, headache, myalgia), and at least one respiratory symptom (cough, sore throat, shortness of breath), and in the absence of other suspected clinical diagnoses.

In the vaccine effectiveness study, we included all patients with available laboratory results and vaccination status recorded in the surveillance system during the study period. Cases were patients laboratory confirmed for pandemic influenza A(H1N1)2009 virus. Controls were patients testing negative for any influenza virus (test-negative controls).

Variables collected for surveillance purpose included: age, sex, clinical symptoms, date of symptom onset, date of swabbing, vaccination status for both pandemic and seasonal vaccines, and laboratory data (confirmation, influenza type/subtype, strain). For the first time, surveillance data also included information on clinical symptoms, chronic conditions and pregnancy. Patients were defined as having at least one chronic disease if they suffered from one of the following conditions: diabetes mellitus, cardiovascular diseases, chronic pulmonary diseases, and congenital or acquired immunodeficiency. Vaccination status (pandemic and seasonal) was collected as a dichotomous variable (yes/no). Data were reported weekly by each sentinel physician to the Spanish region epidemiology unit and included into a web-based application (http://vgripe.isciii.es/gripe).

### cycEVA study

Seven out 17 Spanish regions participated in this study. We invited all 304 sentinel physicians from these regions and 235 (77%) agreed to participate. The population covered by these sentinel physicians represented 2.0% of the total population of the seven Spanish regions.

The 235 study sentinel physicians also participated in the influenza surveillance system and systematically swabbed patients throughout the study period. The definition of cases and controls was the same as in the surveillance-based study. Thus, cases and controls of cycEVA study were also included in the surveillance-based study.

Using a standardised questionnaire, the participating physicians collected in addition to surveillance data (see above) the following variables: date of vaccination, influenza vaccination in the two previous seasons, smoking, functional status, obesity, hospitalisations for chronic conditions, and outpatient visits in the previous 12 months. "Any chronic condition" variable was defined the same as in the surveillance-based study. We defined obesity (body mass index over 30) and pregnancy as risk factors for pandemic influenza. The low functional status was defined as need of help for bathing or walking. A patient was considered vaccinated if he/she had received the pandemic influenza vaccine 14 days or more before the date of symptom onset. Vaccination status was verified against the vaccination registry and/or the patient clinical history available at sentinel physicians' offices.

We excluded from the cycEVA study: patients who refused to participate; those not eligible for influenza vaccination because they suffered from a condition listed in the summary of product characteristics; institutionalised patients; those unable to give informed consent or having received antiviral treatment at the moment of swabbing.

In both studies, we included in the analysis all swabbed patients with less than eight day delay between symptom onset and swabbing and who were attended by sentinel physicians two weeks after the start of the pandemic vaccination campaign (week 48/2009) up to week 8/2010 when the last confirmed influenza A(H1N1)2009 case was reported to cycEVA study. In a subsequent stage, we restricted the analysis to patients meeting the EU ILI case definition. We also estimated the pandemic vaccine effectiveness restricting the analysis to patients with less than four day delay between the symptom onset and swabbing to avoid misclassification due to period of viral shedding.

We calculated the crude and adjusted odds ratios (OR) and their corresponding 95% confidence intervals (95% CI) using logistic regression. We included in the logistic regression models all variables available and known from the literature to influence the influenza vaccine effectiveness estimates as well as the month of swabbing. Thus, the model for the surveillance-based study included: age group, sex, seasonal vaccination, chronic conditions, pregnancy, and month of swabbing. In the cycEVA study, we first adjusted for the variables common to both studies and then we included the additional covariates collected as well as the month of swabbing (full model). We computed PIVE as (1-OR)*100. STATA/IC 10 was used for all statistical analyses (Stata Statistical Software: Release 10. StataCorp LP, TX, USA).

The network-affiliated laboratories or the National Centre of Microbiology (WHO National Influenza Centre) confirmed influenza cases using real-time polymerase chain reaction (PCR) or culture on Madin-Darby canine kidney (MDCK) cell lines. For a proportion of laboratory confirmed cases amplified products were sequenced at the National Centre of Microbiology. Phylogenetic analysis was done to identify the influenza A(H1N1)2009 virus.

Both studies were carried out in the frame of the existing Spanish Influenza Sentinel Surveillance System at the National Centre of Epidemiology; no ethical approval is required for surveillance activities in Spain. However, no personal data were collected and patients gave verbal consent to be swabbed. The cycEVA study was carried out following a generic protocol developed for ECDC [18] and adapted for Spain by the National Centre of Epidemiology.

## Results

**Figure 1:**
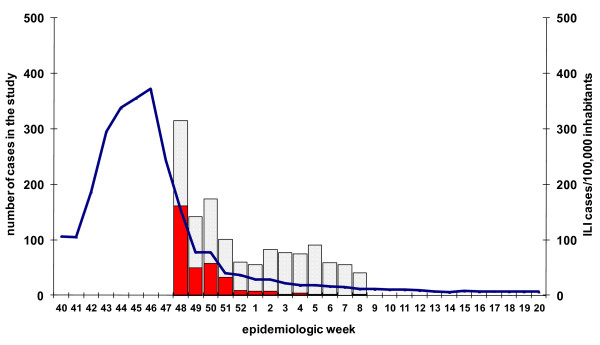
**Surveillance-based study ILI cases (N = 1326) and ILI incidence at national level, season 2009-2010, Spain**. Pandemic influenza laboratory positive cases (red), laboratory negative controls (grey grid) and Influenza-like illness incidence per 100,000 inhabitants at national level (blue line) by week of swabbing in the surveillance-based study.

In Spain, the pandemic influenza activity exceeded the baseline level in the week 40/2009 reaching the peak in the week 46/2009 (372 ILI cases/100,000 inhabitants) (Figure 1). The epidemic period lasted for 11 weeks, from week 40/2009 to week 50/2009. The highest incidence was recorded in the age group 5-14 years old (cumulative incidence: 7507 ILI cases/100,000 inhabitants). The maximum influenza weekly incidence in this age group was 1104 ILI cases/100,000 inhabitants in the epidemic peak.

### Surveillance-based study

From week 48/2009 to week 8/2010, sentinel physicians notified 4580 ILI cases of which 1909 (42%) were swabbed. After excluding patients with no laboratory results (160 records), those with influenza type B or C (six records), subtype H3N2 (two records) or unavailable subtype (seven records), 425 pandemic influenza A(H1N1)2009 cases were recorded. The pandemic vaccination status was available for 1413 (74%) patients. We included in the analysis the 1326 patients swabbed less than eight days after the symptom onset: 331 influenza A(H1N1)2009 cases and 995 test-negative controls. The number of recruited pandemic influenza cases decreased by week of swabbing during the study period (Figure 1). Cases and test-negative controls did not differ by patient characteristics available in the surveillance system, except for clinical symptoms. Cases were more likely to present with sudden onset, fever, malaise, headache, cough and sore throat than controls. Nine cases (2.7%) and 41 controls (4.1%) were vaccinated with the monovalent pandemic vaccine (Table 1).

**Table 1:** Characteristics of influenza laboratory confirmed cases (n = 331) and test-negative controls (n = 995) in the surveillance-based study^a^, season 2009-2010, Spain

Characteristics	Cases n (%)	ILI negative controls n (%)	**p value**^**b**^
Mean age (SD^c^)	23.4 (17.8)	25.4 (21.4)	0.125

Sex: male	162/330 (49.1)	492/984 (50.0)	0.775

Pandemic influenza vaccination	9/331 (2.7)	41/995 (4.1)	0.234

Seasonal influenza vaccination	37/330 (11.2)	104/988 (10.5)	0.727

Symptoms:			
▪ sudden onset	211/241 (87.5)	562/817 (68.8)	**< 0.0001**
▪ fever	321/328 (97.9)	890/991 (89.8)	**< 0.0001**
▪ malaise	268/315 (85.1)	713/967 (73.7)	**< 0.0001**
▪ headache	251/320 (78.4)	588/964 (61.0)	**< 0.0001**
▪ myalgia	213/322 (66.2)	589/978 (60.2)	0.057
▪ cough	295/329 (89.7)	775/985 (78.7)	**< 0.0001**
▪ sore throat	224/322 (69.6)	601/970 (61.9)	**0.013**
▪ shortness of breath	27/305 (8.9)	84/948 (8.9)	0.996

At least one chronic condition	39/247 (15.8)	120/826 (14.5)	0.624

Pregnancy	5/329 (1.5)	7/976 (0.7)	0.187

Complying with EU case definition	195/331 (58.9)	506/995 (50.8)	**0.011**

Delay onset-swabbing less than four days	301/331 (90.9)	874/995 (87.8)	0.124

^a ^Cases and controls recruited between week 48/2009 and week 8/2010, and with a delay symptom onset-swabbing less than eight days; ^b ^Chi-square or Fisher exact test when appropriate; ^c ^SD-standard deviation.

The crude pandemic influenza vaccine effectiveness was 35% (95% CI: -38; 73) and the adjusted value was 62% (95% CI: -5; 87). We obtained similar results in the analysis restricted to patients with a delay symptom onset-swabbing less than four days (Table 2). The adjusted point estimates using patients complying with the EU ILI case definition was 41% (95% CI: -95; 82). When we additionally restricted the analysis to ILI patients swabbed in less than four days since the symptom onset, adjusted PIVE was 48% (95% CI: -110; 82) (Table 2).

**Table 2:** Pandemic influenza vaccine effectiveness in the surveillance-based and cycEVA studies^a^, season 2009-2010, Spain

		Surveillance-based study		cycEVA study	
	Included population	N	**PIVE % (95%CI**^**b**^**)**	N	**PIVE % (95%CI**^**b**^**)**
**Crude**	All patients	1326	35 (-38; 73)	436	58 (-81; 95)
	▪ Delay onset-swabbing less than four days	1175	35 (-45; 74)	381	57(-87; 95)
	EU case definition	701	14 (-131; 72)	377	53 (-109; 95)
	▪ Delay onset-swabbing less than four days	627	5 (-161; 70)	336	64 (-104; 95)

**Adjusted models**	All patients				
	▪ full model^c^			301	75 (-293; 98)
	▪ SISSS covariates^d^	993	62 (-5; 87)	351	65 (-221; 96)
	
	All patients and delay onset-swabbing less than four days				
	▪ full model^c^			258	77 (-296; 98)
	▪ SISSS covariates^d^	853	58 (-21; 85)	302	67 (-211; 97)
	
	EU case definition				
	▪ full model^c^			255	71 (-402; 98)
	▪ SISSS covariates^d^	644	41 (-95; 82)	299	59 (-300; 96)
	
	EU case definition and delay onset-swabbing less than four days				
	▪ full model^c^			231	72 (-290; 99)
	▪ SISSS covariates^d^	568	48 (-110; 82)	263	68 (-215; 97)

^a ^Cases and controls recruited between week 48/2009 and week 8/2010 and with a delay symptom onset-swabbing less than eight days; ^b ^CI = confidence interval; ^c ^Adjusted for age group, sex, seasonal vaccination, chronic conditions, previous hospitalizations, number of GP visits in the previous year, functional status, smoking, previous influenza vaccination, risk factors for pandemic influenza, and month of swabbing; ^d ^Adjusted for age group, sex, seasonal vaccination, chronic conditions, pregnancy, and month of swabbing; SISSS = Spanish Influenza Sentinel Surveillance.

### cycEVA study

**Figure 2:**
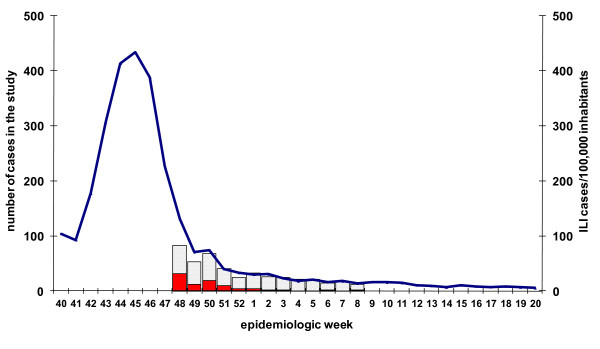
**cycEVA study ILI cases (N = 436) and ILI incidence in the participating regions, season 2009-2010, Spain**. Pandemic influenza laboratory positive cases (red), laboratory negative controls (grey grid) and Influenza-like illness incidence per 100,000 inhabitants in the seven participating Spanish regions (blue line) by week of swabbing in the cycEVA study.

A total of 440 ILI patients were recruited between week 48/2009 and week 8/2010. After excluding cases confirmed for other influenza viruses (one case type B), those with a delay between symptom onset and swabbing of more than seven days (three records), we included in the analysis 436 ILI cases: 85 influenza A(H1N1)2009 cases and 351 test-negative controls. The number of recruited patients decreased during the study period (Figure 2). Most of the patients (62%) were recruited towards the end of the epidemic period (week 48-50/2009). Cases and test-negative controls were similar for most variables included in the study. Among cases, 92.7% presented cough compared to 80.7% controls (Table 3).

**Table 3:** Characteristics of influenza laboratory confirmed cases (n = 85) and test-negative controls (n = 351) in the cycEVA study^a^, season 2009-2010, Spain

Characteristics	Cases n (%)	Controls n (%)	p value^b^
Mean age (SD^c^)	33.2 (17.9)	35.9 (20.0)	0.256

Sex: male	34/85 (40.0)	174/350 (49.7)	0.108

Pandemic influenza vaccination	2/85 (2.4)	19/351 (5.4)	0.395

Seasonal influenza vaccination	9/84 (10.7)	45/347 (12.9)	0.714

Symptoms:			
▪ sudden onset	80/84 (95.2)	322/342 (94.2)	0.699
▪ fever	83/84 (98.8)	321/337 (95.3)	0.138
▪ malaise	77/79 (97.5)	305/317 (96.2)	0.589
▪ headache	70/78 (89.7)	254/310 (81.9)	0.097
▪ myalgia	63/79 (79.8)	275/316 (87.0)	0.100
▪ cough	76/82 (92.7)	268/332 (80.7)	**0.010**
▪ sore throat	58/81 (71.6)	239/315 (75.9)	0.429
▪ shortness of breath	9/74 (12.2)	23/266 (8.6)	0.371

Previous vaccination	9/78 (11.5)	45/300 (15.0)	0.586

Smoking	3/72 (4.2)	18/277 (6.5)	0.586

Any hospitalization	2/75 (2.7)	5/279 (1.8)	0.643

GP visits > eight in the previous year	16/74 (21.6)	68/282 (24.1)	0.653

Any chronic conditions	6/83 (7.2)	53/332 (15.9)	0.052

Pregnancy and obesity	7/84 (8.3)	26/348 (7.5)	0.819

Poor functional status	1/80 (1.3)	18/337 (5.3)	0.143

Complying with EU case definition	77/85 (90.6)	300/351 (85.5)	0.215

Delay onset-swabbing less than four days	80/85 (94.1)	301/351 (85.8)	**0.037**

^a ^Cases and controls recruited between week 48/2009 and week 8/2010 and with a delay symptom onset-swabbing less than eight days; ^b ^Chi square or Fisher exact tests when appropriate; ^c ^SD-standard deviation.

We identified two (2.4%) cases and 19 (5.4%) controls vaccinated with the monovalent pandemic vaccine. The crude PIVE was 58% (95% CI:-81; 95) and that adjusted for all covariates was 75% (95% CI:-293; 98). The PIVE adjusted for the covariates available in surveillance system was 65% (95%CI: -221; 96). We obtained similar results in the analysis restricted to patients with a delay between symptom onset and swabbing less than four days (Table 2). Using the cases that complied with the EU ILI case definition, the adjusted PIVE for all covariates was 71% (95%CI: -402; 98) and 59% (95%CI: -300; 96) when adjusting for the covariates common to both studies. PIVE estimates in the restricted analysis to a delay symptom onset-swabbing less than four days among those that complied to the EU ILI case definition were 72% (95%CI: -290; 99) using the full model and 68% (95%CI: -215; 97) adjusting for covariates available in the surveillance system (Table 2).

### Laboratory findings

Among the 331 influenza A(H1N1)2009 cases reported to the surveillance system during the study period, 66 (20%) were genetically characterised. In the cycEVA study, 68 (80%) specimens were sent for genetic characterisation and 55 (81%) presented sufficient genetic material for the test to be performed. Sequence analysis of the product of amplification (948 nucleotides of the HA1 fragment of the hemagglutinin gene) showed that all the pandemic influenza A strains studied were similar to the vaccine strain A/California/07/2009.

## Discussion

Our results showed a possible protective effect of the pandemic vaccine against medically attended laboratory confirmed pandemic influenza A(H1N1)2009 in Spain, in the context of low vaccination coverage and low ILI incidence at the end of the epidemic period when the study was carried out. However, these results are consistent with the good matching between the vaccine and circulating strains.

Only a few vaccine failures were recorded in both studies. Pandemic vaccination started during the epidemic peak in the week 46/2009, leaving only a short period of time to enable vaccine effectiveness studies (after week 48/2009). In addition, the vaccination coverage was lower than expected, reaching 16% in the target groups for vaccination at the national level [19]. The pandemic vaccination coverage in the sentinel physicians' catchment area of the seven participating networks was 10.7%. This made difficult to estimate with precision the PIVE in both studies.

We adjusted for most of the confounders described in the literature in the cycEVA study and for available confounders in the surveillance-based study. The covariates that mainly influenced the results (more than 5% difference between the crude and Mantel-Haenszel adjusted OR) were: chronic conditions and seasonal vaccination 2009-2010 for the surveillance-based study and age, sex, chronic conditions, month of swabbing, functional status, at least one hospitalisation in the previous 12 months, and GP visits in the previous 12 months for the cycEVA study (data not shown). cycEVA PIVE estimates using the full model were around 10% higher than the PIVE adjusted only for the variables collected in surveillance system. When adjusting for the common variables in the surveillance-based and cycEVA studies, the PIVE point estimates were similar for all patients. Restricting the analysis to a delay symptom onset -swabbing less than four days or to patients meeting EU case definition, the PIVE was around 10-20% higher in the cycEVA study, probably reflecting the better data collection in this study. Nevertheless, the point estimates in both studies are lower than those reported by other authors [20-24]. The differences may be related either to the different outcomes and study designs or to the different confounding factors adjusted for. A European multicentre study (I-MOVE) in which Spain participated with data from the cycEVA study confirmed the cycEVA estimates by pooling data from seven countries [25].

The results of both studies suggest the presence of negative confounding [26], because the adjusted point estimates were higher than the crude ones. When we measured the effectiveness of the seasonal vaccine during the previous influenza season [6,11], the adjusted VE estimates were lower than the crude VE, suggesting the predominance of positive confounding. Because the Spanish population consults GPs more often than the EU average (outpatient contacts/person/year 9.5 in 2003 in Spain and 7.67 in the EU) [27], we believe this increases the probability that more elderly people with better functional status are attended by the sentinel physicians (healthy vaccinee effect). This was not the case during the 2009-2010 season. The observed negative confounding might be related to the dynamics of the influenza activity and/or to different health seeking behaviour during the pandemic in Spain. Further studies are needed in the future influenza seasons to better understand these findings.

Among the limitations of the surveillance-based study, we should also mention the lack of collecting of the pandemic vaccination date. Unlike the usual influenza seasons when the vaccination campaign complete before the epidemic period, the pandemic vaccination started and was carried out during the pandemic wave. In this context, determining the delay between vaccination and symptom onset became of crucial importance because some individuals classified as vaccinated may not have been protected, resulting in an underestimation of the vaccine effectiveness. However, 80% of pandemic vaccinations were performed in the first two weeks of the campaign [source: Ministry of Health and Social Policies, Spain, unpublished data]. Restricting the study period to two weeks after the start of the vaccination campaign might have reduced this misclassification in the surveillance-based study.

As it was impossible to distinguish the cycEVA patients in the surveillance database, we were not able to compare cycEVA patients to the rest of the surveillance system. However, subtracting the Spanish regions participating in cycEVA from the total number of patients included in the surveillance study, the crude OR was 0.83 (95%CI: 0.29; 2.04), resulting in a crude pandemic vaccine effectiveness of 17%. This might be related to a lower compliance to the EU ILI case definition in the rest of the surveillance system compared to cycEVA or to a higher weighting given to the clinical judgement during the pandemic season due to the additional statement "in the absence of other suspected clinical diagnoses" added to EU ILI case definition. In fact, sentinel physicians participating in the cycEVA study used the mentioned EU ILI case definition since the season 2008-2009, meanwhile the rest of surveillance system adopted it for the pandemic 2009-2010 season. This was only revealed collecting the clinical symptoms in the surveillance system which allowed estimating the compliance with the EU ILI case definition. We also found that more than 40% of influenza laboratory confirmed cases notified to the surveillance system did not meet the EU ILI case definition (low sensitivity), suggesting that the clinical judgement of the sentinel physicians might be useful for the recruitment of true positive cases. These findings underline the need for further studies related to EU ILI case definition.

The well established influenza sentinel surveillance system in Spain rapidly adapted to monitor pandemic influenza and allowed studying different aspects of its activity, including vaccine effectiveness. Data were weekly notified and analysed at regional and central level. Several improvements were implemented in the surveillance system for the pandemic season: data collection was expanded to include clinical symptoms, chronic conditions and pregnancy; systematic swabbing was introduced. On the other hand, cycEVA study presented an added value for the surveillance system. Firstly, additional information was collected on confounding factors known from the literature to influence influenza vaccine effectiveness estimates. Secondly, collecting the date of vaccination allowed avoiding misclassification of those vaccinated but not protected, which proved to be essential during the pandemic season. Last but not least, the data collection in the cycEVA study was more complete and periodically validated.

## Conclusions

The sentinel influenza surveillance system in Spain allowed estimating the pandemic influenza vaccine effectiveness during a season with a high sentinel physicians' workload. The results of both studies suggested a possible protective effect of the vaccine against laboratory confirmed influenza A(H1N1)2009 infection, higher when vaccination date is collected and some important confounding factors are taking into account. This suggests that the routine influenza surveillance system provides reliable estimates and could be used for influenza vaccine effectiveness estimates, if the data collection is improved by taking into account the date of vaccination and some additional potential confounding factors.

The pandemic season 2009-2010 represented a challenge for vaccine effectiveness studies due to the availability of the vaccine after the pandemic peak and the low pandemic influenza vaccination coverage. Repeating both studies in future seasons and using cycEVA study as a validation subset of the whole surveillance system will provide further information to draw conclusions on the effectiveness of the influenza vaccination in Spain.

## Competing interests

The authors declare that they have no competing interests.

## Authors' contributions

CS analysed and interpreted both cycEVA and surveillance-based data and wrote the first draft of the article. AL, SM, SJJ, FP analysed the surveillance data, participated in data analysis and interpretation for both cycEVA and surveillance-based studies and revised all drafts of the manuscript. FP, IC, PPB, AG, JMV, CR, TV, AM, NT, JMR, MCS, JC, MGC, JMAl, JMAr, CQ, MP collected data of the cycEVA study and participated in the interpretation of the data. All authors revised and approved the final draft. CS and AL are equally responsible for this article.

## Pre-publication history

The pre-publication history for this paper can be accessed here:

http://www.biomedcentral.com/1471-2458/11/899/prepub
